# Improving farm households’ economic status to address food security in Ghana: The role of participation in nonfarm activities

**DOI:** 10.1016/j.heliyon.2025.e42496

**Published:** 2025-02-05

**Authors:** Eli Andrew Akosikumah, Hamdiyah Alhassan, Paul Adjei Kwakwa

**Affiliations:** a Department of Agricultural and Food Economics, University for Development Studies, Tamale, Ghana; b Department of Economics, University for Development Studies, Tamale, Ghana; c School of Arts and Social Sciences, University of Energy and Natural Resources, Sunyani, Ghana

**Keywords:** Security, Nonfarm, Employment, Household, Sustainable livelihood

## Abstract

Understanding highly pragmatic ways of improving the quality of life of farming households by way of ensuring food security among these households has been a topical issue among many researchers. Closely linked to this technique is the participation in nonfarm activities. For a farming household, participation in such activities serves as a hedge against the risk associated with farming and by far a potential for addressing food insecurity. This study assesses the impact of participation in formal and informal nonfarm activities on household food security in Ghana using data from the Ghana Living Standard Survey 7 (GLSS 7). Multinomial Endogenous Treatment Effect was used to estimate the determinants of households' decision to engage in nonfarm activities and household food security. PSM was further used to evaluate the impact of participation in nonfarm activities on household food security. Overall, the study documents that participation in formal and informal nonfarm activities increases per capita consumption expenditure by 10 % and 9.7 % respectively. Nonfarm participation is a sure means of reducing household food insecurity shocks and improving household wellbeing. Policymakers in Ghana should therefore pay attention to nonfarm activities as a means of improving households’ welfare.

## 1 Introduction

Food insecurity is pervasive within Africa where more than half (799 million) of its population is faced with moderate or severe food insecurity with additional 347 million severely food insecure [1]. Across sub-Saharan Africa, a substantial fraction is considered food insecure at national, household and individual levels using total per capita caloric intake [2]. This poses as a hindrance in achieving the universal SDGs 1 and 2 of eradicating poverty and hunger. Thus food security seems to promote hunger reduction, improve nutrition and long-term economic growth [3].

Although, Ghana has attained the Millennium Development Goals (MDGs) of halving poverty and ensuring food security, Ghana still has segments of the populations that are food insecure which has attracted the attention of many [4,5]. Almost 1.2 million Ghanaians are food insecure with extra 2 million people reportedly vulnerable to food insecurity [6,7]. The dynamics of poverty in Ghana shows that poverty and food insecurity levels in Ghana are rural phenomenon and more prominent among farmers [6,8]. It is also high among farming-owning households than non-farm-owning households [9]. Policies in the agricultural sector are geared towards increasing the incomes of the poorest farmers and minors while improving their food security status, yet the incomes of majority of farmers remain low exposing them to various forms of food insecurity and poverty [10].

To many policymakers, researchers and development practitioners, the assertion of investing in farming activities seems obvious to improving welfare of farmers. Thus, major policies in improving livelihoods of farmers in many developing countries have centered on increasing productivity through intensive input usage. For instance, Ghana Government's Planting for Food and Jobs, and Rearing for Food and Jobs are underscored by improving food security for farming families through intensive use of conventional inputs [11]. Unfortunately, farmers in developing countries rely on traditional methods of production which reduce their productivity level [12]. Again, with the recessive agriculture characterized with unstable weather conditions, persistent drought, incidence of diseases and pests, high population growth, weak institutional capacity and inadequate infrastructures [13,14] dependence on farming alone cannot serve as a number one panacea in alleviating poverty and tackling food insecurity. Consequently, farmers in these countries are trapped in cyclical food insecurity and poverty owing to their dependence on rain-fed agriculture, a situation that has made farmers a priority for development efforts [[15], [16], [17], [18], [19]].

A complementary concern to small scale faming is the uptake of nonfarm economic activities to augment income from farming. A fundamental factor of household food security accessibility which can be realized through income. Studies have demonstrated that nonfarm activities are important in ensuring access to food, reduction in malnutrition and consumption smoothening thereby reducing households' vulnerability to food insecurity, bridging poverty gap and ensuring equitable income distribution among households [[20], [21], [22], [23], [24], [25], [26], [27], [28]]. Nonfarm activities further minimizes the movement of many rural dwellers to urban centers by providing job opportunities. Hence, these activities can be viewed as a viable strategy for poverty reduction [29,30]. Engaging in nonfarm activities can complement farm productivity through the investment of nonfarm income into farming businesses by purchasing farm inputs [[31], [32], [33]] thus reducing farming households’ vulnerability to food insecurity.

Some scholars [[34], [35], [36], [37], [38], [39], [40], [41]] have shown interest in livelihood diversification of households in Ghana. However, these studies and others outside Ghana [10,20,21,[42], [43], [44], [45]] usually do not distinguish the effect of formal and informal nonfarm activities. However, participation in formal and informal nonfarm economic activities can have varied effects on household welfare. For instance Rammohan and Vu [46] have indicated that although nonfarm income improves food security, informal employers who are more likely to be underpaid, receive lower income and low job security, their food expenditure may be lower and as such be more food insecured than their counterparts in the formal sector who may receive higher income and have their job secured. Blekking et al. (2019) have also suggested that within the African context it is possible for those in the informal sector to earn similar wages as those in the formal sector and as such have high food security. From the above argument, given that the informal sector in Africa and for Ghana in particular remains significant in terms of offering job opportunities to a large number of people who perhaps could not be absorbed by the formal sector [47] analyzing their respective effect on farm households food security is significant for the country. Again, studying the distinct impact of formal and informal nonfarm activities will ensure that welfare and developmental policies are tailor-made to correspond to specific intervention to achieve desired targets. Thus, analysis that will distinguish the effect of the two will be appropriate for better policy formulation. Also in the case of many developing countries although studies on household livelihood diversification abound [[48], [49], [50]] there is limited knowledge on what drives farmers to engage in formal and informal nonfarm activities. Identifying the influential factors of farm households to engage in formal and informal nonfarm activities is essential in at least two ways: a) studies on drivers of livelihood diversification have yielded conflicting results which calls for more contextual studies. More so, a study that focuses on factors affecting livelihood diversification based on the sectors will help throw more light on the issue in the literature. b) it will also offer insightful information to policymakers and others who may want to guide farmers diversify their livelihoods based on their conditions.

From the above the objective of this study is to explore the food security effect of farm households’ participation in formal and informal nonfarm activities in Ghana employing the Multinomial Endogenous Treatment Effect (METE) regression. The specific objectives are:

a) to identify the determinants of participation in nonfarm activities in Ghana;
b) to examine the determinants of household food security in Ghana; and
c) to investigate the impact of participation in nonfarm activities on food security in Ghana.

The contributions from this study are in three folds. First, the study offers evidence from an African country on the drivers of participation in nonfarm activities and farm household food security. Second, the effect of formal and informal nonfarm activities on food security is examined. Third, in terms of methodology, the study employs the Multinomial Endogenous Treatment Effect (METE) regression in assessing the determinants of nonfarm participation and food security. This method correct for potential problem of selectivity bias due to observed and unobserved factors. Finally, it also employs recently available data.

## 2 Literature review

The idea of nonfarm activities comprises of activities that are directly beyond agricultural production and thus involve industry, manufacturing and trade [25,51]. These activities consist of traditional pottery, weaving, tailoring, modern production and distribution of community and personal services [52]. Rural nonfarm activities play a vital role in transforming the economies of many developing nations by serving as a strategic way to respond to shocks resulting from climate change [53]. It appears nonfarm activities have become a common feature in both developed and developing economies due to the low level of income among farm households [54]. In addition, the vulnerability associated with farming compels many farmers to engage in nonfarm activities to diversify their income source. Meanwhile, it is also mentioned that limited skills, financial constraints and lack of specialization are among the reasons that impede some farm households from engaging in nonfarm activities [55].

Econometric estimation by Iqbal et al. [54] revealed that farmers engage in nonfarm activities for reasons such as low income from agriculture, family burden, desire to work on something else, reducing income risk from farming, and availability of off-farm activities. Assessment to ascertain the influence of socio-economic factors on nonfarm activities revealed farm size, active work, access to roads, dependency ratio, years in farming and household size significantly increases the likelihood of nonfarm engagement in Pakistan [54]. In Lesotho, Lelimo et al. [56] obtained positive effects of gender, education of household heads and household size on nonfarm engagement. Duong et al. [57] also estimated that marital status, male and farm size reduce farmers engagement in wage employment while number of adults and loss due to agricultural shocks increase the tendency for farmers in Vietnam to engage in nonfarm employment. Lim-Applegate et al. [58] had earlier estimated among Australian households and observed that age has inverted U-shape relationship with nonfarm activities. Also, while education positively influence nonfarm employment, the presence of preschool children reduces the tendency. In Ecuador, Vasco and Tamayo [59] reported that access to electricity and telephone, wealth, female adults, female head and education increase participation in nonfarm. Similarly, land size, age, household size, access to credit, distance from home to the nearest market center, special skill, access to electricity and access to irrigation influences Ethiopian farmers decision to participate in nonfarm work [60].

Participation in nonfarm activities is substantially important in raising household food security mainly in rural households (food expenses, dietary diversity, share food expenses, food accessibility, food utilization and stability) through increased income and more productivity. Empirical studies demonstrated that a 10 % increase in income from nonfarm activities increases household food adequacy by 0.3 % [61]. Ackah [34] concluded that participation in nonfarm employment significantly increases household income in Ghana. Increase in income is therefore expected to help reduce food insecurity of farmers. The findings of Osarfo et al. [44] points out that nonfarm engagement have positive impact on the food security and household income of farm households in rural Ghana. Similarly, Owusu et al. [40] reported that households who participate in nonfarm activities have a higher income. Tesgera et al. [20] and Séogo [21] reported that participation in non-farm activities increase household consumption per capita in Ethiopia and Burkina Fasso respectively. In Nigeria, Shehu and Sidique [45] employed propensity score matching to analyze the impact of nonfarm enterprise engagement on household consumption expenditure. The study found that engagement in nonfarm activities by households increased household consumption expenditure. Mondal et al. [31] found in their study in Bangladesh that nonfarm income ensures agricultural intensification by reducing liquidity constraints faced by farmers, consequently increasing agricultural production. Once productivity increases it has a higher chance of reducing food shortage. Ba et al. [55] also reported that nonfarm activities reduce farm households’ poverty thereby enabling them to meet their food needs. The findings of Duong et al. [57] indicated that nonfarm employment improves income and food security thereby contributing to poverty alleviation. In Cambodia, Do et al. [62] revealed that food accessibility, utilization, and stability were affected by nonfarm activities. Zerai and Gebreegziabher [60] found that nonfarm employment provides extra income that helps Ethiopian farmers to increase their expenditure on food, clothing and health care. Other factors that have been found to positively influence household food security are family size, land ownership and land size [60]. Rahman and Mishra [63] found that in India non-agricultural income increases overall expenditure on food, particularly on non-cereal items thereby ensuring higher dietary diversity. Also, benefit from government and remittance were found to increase food expenditure.

In his work on rural Cambodia, Seng [64] obtained a positive effect of nonfarm income on food consumption. It was also reported that education of household head and landholding increase food security while household size reduces food security. Dzanku [37] found for a group of African countries that nonfarm income reduces food insecurity by increasing food availability. Female headed households were also found to positively affect food security among the countries. In a study on Nigerian farm households, Ojeleye et al. [65] recorded household size, consumer credit and farm income reduce food security while nonfarm income, crop output and farm size increase food security.

## 3 Methodology

### 3.1 Data source

Secondary data sourced from the Ghana Statistical Service was used for the study. The data which is a cross-sectional nationally representative data was collected by the Ghana Statistical Service with technical and financial support from the Government of Ghana (GoG), United Kingdom Department for International Development (DFID), the Dutch Government and the World Bank. More specifically, we used data on farming households from the Ghana Living Standard Survey Round 7 collected in 2016-17. The GLSS7 is a comprehensive household data collected across the entire country (Ghana) focusing on different demographic indicators of living conditions spanning from education, health, agriculture and other economic activities, migration, tourism, housing, food security and household food and non-food expenditure. Data collection was carried out in a very systematic approach to ensure nationally and regionally representative information. The data covered 14, 009 households selected from 1000 enumeration areas.

**Fig. 3.1:**
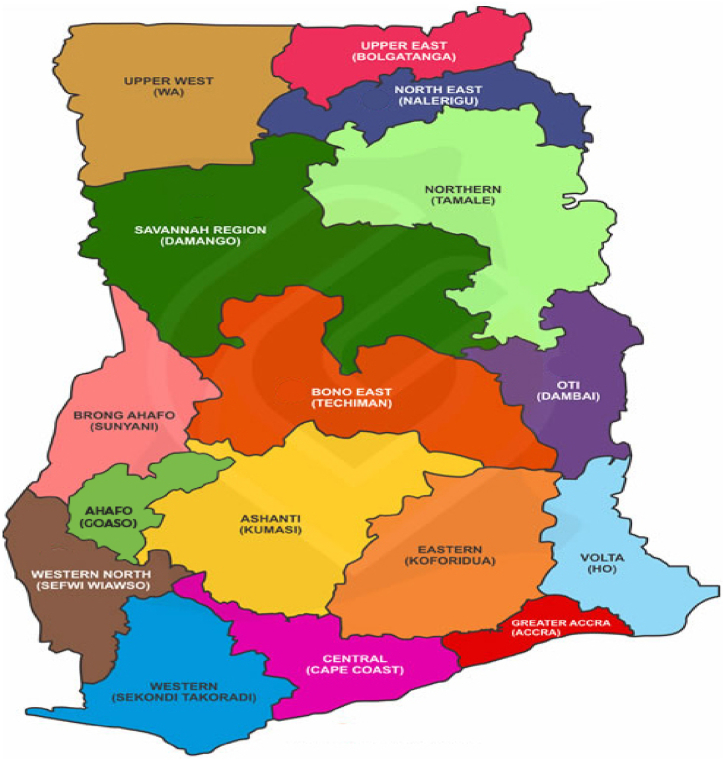
Map of Ghana.

Source: https://www.ghanamissionun.org/wp-content/uploads/2023/11/ Ghana_Regional_Map.jpg

For this study, the selection of respondents followed the following processes: In the first place, based on the objective, the study restricts the sample to farming households who undertake either formal and/or informal non-farm economic activity. Again, the analysis focused on farming households that cultivated between 0.5 and 50 acres. This was done to exclude farm households with less than 0.5 acres of agricultural land that engaged in either urban or rural vegetable gardening. Similarly, farming households that cultivated more than 50 acres were further excluded in order to exclude large-scale farmers. These processes gave us a sample of 4,120 observations. The treatment categories consist of households engaged in farming only (3421), farming and informal non-farm work (570) and farming and formal non-farm work (129). Fig. 3.1 shows the map of the study area.^1^

### 3.2 Theoretical framework

The study fits itself within the theory of time allocation and utility. A farming household's decision to engage in nonfarm business can be explained by theory of time allocation and utility [66,67]. A key feature of the theory is, farmers choose an economic activity that give them optimum utility conditional on the constraints affecting their decision, particularly time, labour [66]. Again, the choices household make depends on both household specific and unobserved characteristics. When a household's intra-household allocation of time is exogenously given, the utility maximization function is subject to total time (T) available for farming or farming combined with nonfarm business, income (I) and the quantity (Q) of agricultural products. This is expressed mathematically in equations (1), (2):

(1) maxU(Participationineconomicactivity)

(2) subjectto(T,I,Q)

Generally, two aspects of time (T) is available for households (24 h); thus time allocated for economic activity/work $\left(T_{e}\right)$ and time available for leisure $\left(T_{l}\right)$. For a typical farming household, time available for work $\left(T_{e}\right)$ can be expanded to include time allocated for farming $\left(T_{f}\right)$, formal nonfarm ($T_{e}^{f}$) and/or informal nonfarm activity($T_{e}^{i})$. This is expressed in equation (3) below,

(3) $$T=T_{f}+T_{e}^{i}+T_{e}^{f}+T_{l}\;\text{where}\;T_{e}^{i}+T_{e}^{f}\geq 0$$

Total household income is systematic and implicitly determined by income from agriculture ($I_{ag}$), wage from nonfarm economic activities ($W_{t}$), remittances (R) and other form of income such as bonuses and retirement benefits (B). Equation (4) presents the mathematical expression for total household income.

(4) $$I=I_{ag}+W_{t}+R+B$$

The third crucial constrain to a household's utility maximizing function is the quantity of output produced. The production of total agricultural output (Q) therefore depends on the heterogeneous inputs (X), production technology (J), household labour for farming activities (L), human capital endowment (H) and the farming environment determined by the agro-ecological conditions and weather parameters (Y) (Gebremariam & Wünscher, 2016; [68]). The function for the total output (Q) produced is expressed in equation (5):

(5) Q=f(X,J,L,H,Y)

Netting out the cost of inputs whiles placing restrictions on savings, the income from agriculture equation can be equated to the quantity of farm output; which is presented as equation (6).

(6) $$I_{ag}=Q=X,J,L,H,Y$$

Households’ utility maximizing function subject to their constrains can be specified as equation (7);

(7) $$U=T_{f}+T_{e}^{i}+T_{e}^{f}+T_{l}\;;\;f\left(X,J,L,H,Y\right)+W_{t}+R+B$$

with the condition for maximization expressed in equation (8).

(8) $$\int^{!}{\;>\;0;\int }^{!!}\;<\;0$$

### 3.3 Econometric modelling and estimation strategy

The paper applied the Multinomial Endogenous Treatment Effect (METE) regression in assessing the determinants of nonfarm participation and food security. The model intrinsically uses a maximum likelihood approach or a two-step control function under joint normality [69,70]. For the impact estimation process, two major equations were identified. The treatment module and the outcome module.

The treatment module comprises three set of binary choices, namely;

i. Treatment 0 means a farming household participated in only farming 0 (t = 0)
ii. Treatment 1 means a farming household participated in farming and informal nonfarm activities (t = 1)
iii. Treatment 2 means a farming household participated in farming and formal nonfarm activities (t = 2)

These choices are mutually exclusive emphasizing the point that, a household chooses to engage in only one of the combinations of these activities at a point in time. These multiple choices/treatments were modelled using multinomial choice model. Following the specification of Deb and Trivedi [71], ${EV}_{ij}^{*}$ is a latent variable capturing the indirect utility derived by the $i^{th}$ household from consuming the income generated from the $j^{th}$ venture, where j represents the categorical variable describing the choice of an economic activity undertaken by the $i^{th}$ farming household among *m* categories; thus j = 0, 1 and 2. The model capturing the indirect utility term, thus the latent variable is expressed as equation (9):

(9) $$EV_{ij}^{*}=z_{i}^{\iota }\beta_{j}+\sum_{k=1}^{J}\delta_{ik}l_{ik}+\eta_{ij}$$

$Z_{i}$ captures a set of explanatory variables, $\beta_{j}$ signify econometric parameters associated with the exogenous variables, $\eta_{ij}$ is error term that is independently and identically distributed; $l_{ik}$ is the latent factors such as famers innate ability like business mindedness or desire to generate extra income that captures unobserved characteristics associated with both the participation in nonfarm activities and food security indicators (per capita consumption expenditure, household dietary diversity score (HDDS) and Food Consumption Score (FCS) with $\delta_{j}$ vector of corresponding parameters. The model further assumed that the latent factors $l_{ik}$ are independent of the term $\eta_{ij}$. Generally, j = 0 represent farmers who engage in only farming (control group) and ${EV}_{ij}^{*}=0$. Whiles the indirect utility term ${EV}_{ij}^{*}$ is not observed, farmers’ decision to participate in other nonfarm activities can be observe as a set of binary variables $d_{i}$ that captures the observed treatment choice.

Thus $d_{i}=d_{i1},d_{i2,}\ldots \ldots \ldots \ldots \ldots \ldots \ldots d_{ij}$. Also, $l_{i}=l_{i1},l_{i2,}\ldots \ldots \ldots \ldots \ldots \ldots \ldots l_{ij}$. Then the probability of a farming household participating in a given nonfarm activity given a set of exogenous variables can be stated as equation (10):

(10) $$\mathrm{Pr}\left(d_{i}|z_{i},l_{i}\right)=g\left(z_{i}^{1}\alpha_{1}+\delta_{1}l_{i1},z_{i}^{1}\alpha_{2}+\delta_{2}l_{i2},......................\;z_{i}^{1}\alpha_{j}+\delta_{j}l_{i\;j}\;\right)$$

**g** follows multinomial probability distribution. According to Ref. [71], g has a mixed multinomial logit (MMNL) structure that is defined as equation (11).

(11) $$\mathrm{Pr}\left(d_{i}|z_{i},l_{i}\right)=\frac{\exp\left(z_{i}^{1}\alpha_{j}+\delta_{j}l_{i\;j}\right)}{1+\sum_{k=1}^{J}\;\exp\left(z_{i}^{1}\alpha_{k}+\delta_{k}l_{ik}\right)}$$

In the second stage, the impact of nonfarm engagement on the outcome variable y (per capita consumption expenditure, HDDS and FCS) was carried out. The expected outcome for an individual household can be estimated using equation (12):

(12) $$Ε\left(y_{i}|d_{i},x_{i},l_{i}\right)=\left(x_{i}^{\prime }\beta +\sum_{j=1}^{J}\gamma_{j}d_{ij}+\sum_{j=1}^{J}\lambda_{j}l_{ij}\right)$$

$y_{i}$ represent food security indicators (adult equivalent of per capita consumption expenditure, HDDS and FCS) for an individual household i in equation (12); $x_{i}$ represent a set of covariates with its accompanying parameters β. The parameters $\gamma_{j}$ denotes the treatment effects in relation to the base category (Farmers only).

Farmers’ participation in farming and nonfarm activities may not be random. In other words, farmers may self-select themselves in participating in farming and nonfarm activities. The function $E\left(y_{i}|d_{i},x_{i},l_{i}\right)$ is an expression of the latent factors $l_{ij}$ explaining the relationship between the food security indicators and nonfarm engagement as a result of the effect of unobserved characteristics. This will lead to possible endogeneity in the choice of economic activity that would lead to a bias estimate of $\gamma_{j}$ given that $d_{ij}$ is exogenous. It is worth noting that when the factor-loading parameters $\left(\lambda_{j}\right)$, is positive or negative, then there is a negative or positive correlation between nonfarm engagement and food security indicators as a result of unobserved characteristics. In other words, there exist either a positive (negative) selection, with γ and λ the associated parameter vectors, respectively [52,72]. To address this challenge, METE models accounts for this potential bias through a selection correction process by calculating an inverse Mills ratio (IMR) using the theory of latent factor structure [72,73]. Since the food security indicators (adult equivalent of per capita consumption expenditure and FCS) are continuous we follow the normal (Gaussian) distribution function and poisson distribution for HDDS. The model was estimated using Maximum Simulated Likelihood (MSL) method.

According to Deb and Trivedi [71], the estimated parameters of METE can be identified even if $z_{i}$ = $x_{i}$, although the inclusion of some variables in the $z_{i}$ that are not included in $x_{i}$ or the inclusion of at least one instrumental variable for a more robust identification is preferable. Practically, getting a valid instrument is difficult [52,[74], [75], [76]]. The study used ownership of phone as an instrument. With regards to ownership of phone, this was perceived to be real and true considering the fact that household members can use phone to obtain information on nonfarm employment availabilities within and beyond their locations. Empirically, Osarfo et al. [44] has demonstrated that phone is a significant factor affecting household decision to engage in nonfarm activities. The study carried out admissibility of the instrument by performing simple falsification test [74]. According to the test, an instrument is valid when it significantly affect households’ decision to engage in nonfarm activities not significant in explaining household food security status [74]. The result presented in Appendix B, showed that ownership of phone is a valid instrument that affect the treatment variable but did not significantly affect household food security status. Table 1 presents the measurement of the variables adopted for the estimations.

**Table 1:** Summary of Explanatory variables and a priori expectation.

Variable	Measurement
***Outcome variables***	
Household food consumption expenditure	Continuous (adult equivalent of per capita food consumption expenditure)
Food Consumption Score (FCS)	Continuous (Household caloric intake and diet quality)
Household Dietary Diversity Score (HDDS)	Count of the different number of food groups consumed by the households over a reference period of seven days
***Treatment variable***	
Farming	Engaged in farming only
Farming and Informal nonfarm	Engaged in farming and informal nonfarm work
Farming and formal nonfarm	Engaged in farming and formal nonfarm work
**Explanatory variables**	
Location	Dummy: 1 = Urban; 0 = Otherwise
Age	Years
Sex	Dummy: 1 = Male; 0 = Otherwise
Marital status	Dummy: 1 = Married; 0 = Otherwise
Household size	Continuous (Total number of people within a household)
Education	Years in formal education
Farm size	Acres
Ownership of bank account	Dummy: 1 = Own bank account; 0 = Otherwise
Access to credit	Dummy: 1 = Yes; 0 = Otherwise
Remittances	Dummy; 1 = Receipt of remittances; 0 = Otherwise
Shock	Dummy: 1 = Death of a household member; 0 = Otherwise
Total crop value	Continuous (Farmers' estimation of how much (in Ghana cedis) they would have gotten if they were to sell all the harvested crops)
Polygamous family	Dummy: 1 = Polygamous; 0 = Otherwise
Ownership of fridge	Dummy: 1 = Own a fridge; 0 = Otherwise
Ownership of phone	Dummy: 1 = Own a phone; 0 = Otherwise
Total Livestock Unit (TLU)	Continuous (total amount of livestock owned by a household)
Agro- ecological zone Forest zone Savannah zone	Categorical Dummy: 1 = forest zone, 0 = otherwise Dummy: 1 = Savannah zone, 0 = otherwise

Dietary diversity score was measured by counting the different number of food groups consumed by the households over a reference period of seven days [[77], [78], [79]]. The counting process was first carried out by classifying the various foods items consumed by households in the past thirty (30) days of the enumeration period into food groups following the guidelines of FANTA [80]. The classification of the food groups was based on the nutritional characteristics such as energy content, protein, mineral or vitamins level [77]. Following the classification by Kennedy et al. [78], the HDDS for the study was determined from (1) Grains and Flours, (2) Roots, Tubers and Plantain, (3) Pulses, nuts and seed oil, (4) Fruits, Vegetables, (5) Meat, poultry and fish, (6) Other livestock product and (7) Drinks and beverages. The highest frequency obtained was 7 for all the food groups implying that some households consumed from all 7 groups during the past seven days. Food consumption score (FCS) is composite score generated from assigning weights to the frequency of the food groups consumed. FCS captures aggregate household level data on the diversity and frequency of food groups consumed over a reference period often seven days, which is then weighted based on the relative nutritional value of the food groups consumed (INDDEX Project, 2018). We compute FCS using grains and Flours, Roots, Tubers and Plantain, Pulses, nuts and seed oil, Fruits, Vegetables, Meat poultry and fish, other livestock products, Drinks and beverages consumed in the past 7 days. The model used is defined as equation (13).

(13) $$\text{FCS}=\alpha_{\text{grains}}x_{\text{grains}}+{\;\alpha }_{\text{tubers}}x_{\text{tubers}}+\alpha_{\text{pulses}}x_{\text{pulses}}+\alpha_{\text{fruits}}x_{\text{fruits}}+\alpha_{\text{Vegetables}}x_{\text{vegetables}}+\alpha_{\text{meat}}x_{\text{meat}}+\alpha_{\text{beverages}}x_{\text{beverages}}$$

Where.

FCS = Food Consumption Score

***a*** = Weight of each food group

***x*** = Frequencies of food consumption (number of days for which each food group was consumed during the past 7 days)

The weight for staples/grains and tubers base on the energy density and protein content was 2, 4 for meat, fish, milk and milk products. Pulses were assigned a weight of 3, 1 for vegetables and fruits [7].

The explanatory variables employed in the study were measured as follow. Location is referred to as the geographical location of the farming household. It was considered as a dummy variable where urban households are coded 1 and 0 for rural households. Age of the household head was measured in years. Sex of respondents was treated as a dummy variable, male are coded 1 and 0 for female. Marital status was measure as a dummy variable where married household heads were assigned 1 and 0 otherwise. Household size is the total number of people within a household. It was measured as a continuous variable. Farmers level of education was measured in years spent in formal education. Farm size defined as the total farmland cultivated by the household measured in acres. Access to credit has also been treated as a dummy variable (1 if yes, 0 if no). Remittances was treated as a dummy variable; 1 if a households received remittances and 0 otherwise. Shock was defined as the death of a household member in the past 12 months, 1 if a household experience a death of a member and 0 if otherwise.

Crop value was obtained by farmers' estimation of how much (in Ghana Cedis) they would have gotten if they were to sell all their harvested crops. These values were transformed using the natural logarithm in order to induce normality and reduce high variations. This was treated as a continuous variable. Polygamous family was computed using male household heads with more than one wives as 1 and 0 as male household heads with only one wife. The livestock unit (TLU) is an international accepted way for measuring the total amount of livestock owned by a household. The process describes the different species and age of livestock using a commonly accepted unit called the TLU base on the metabolic weight and the nutritional value of the animal species. In estimating the TLU, eight types of livestock were considered, namely; goat, donkey, cattle, sheep, horses, rabbits, pigs and poultry (chicken, guinea fowls, ducks, turkeys, etc.). The computation of the TLU made reference to the Food and Agriculture Organization's livestock convention units developed for the Sub-Saharan Africa. According to the reference a Tropical Livestock Units (TLU) for cattle is 0.50, 0.80 for horses and donkeys, 0.10 for sheep and goats. The weight for ducks was set to be 0.03 for ducks, turkeys and geese, 0.02 for rabbits, 0.20 for pigs and 0.01 for chicken and guinea fowls (FAO, 2003; [81]). Ownership of fridge was measured as a dummy variable where 1 denotes a household owns a fridge and 0 otherwise. Ownership of phone was measured as a dummy where 1 denotes a household owns a phone and 0 otherwise.

## 4 Results and discussions

### 4.1 Descriptive statistics

From Table 2a, Table 2ba and 2b, about 11 % of the farming households were urban households, while most (89 %) were rural households. Majority (79.30 %) of farming households in Ghana are male headed. An average Ghanaian farmer was found to be 48.43 years with an average of about 2 years of educational attainment. The average age of farming household heads reflects the high dominance of youths within the Ghanaian agriculture sector. On average, farming households spent Gh₵ 1,151.92 on formal education. A typical household in Ghana was found to have an average of 5 persons whiles the average farm size cultivated was about 6.77 acres. The mean crop value obtained by farmers was found to be Gh₵ 2, 963.01. It was found that the value of agricultural asset owned by farming households was Gh₵ 333.89. Farming households on the average spent about 33 h on their farm in a week. Also, only 10.5 % of farming households had access to credit for farming activities while approximately 5 % of farming households received remittance. Expenditure on food among Ghanaian farmers was estimated to be (Gh₵ 1,571.123)

**Table 2a:** Descriptive statistics of explanatory variables – categorical variables.

Variable	Percentage	Min	Max
**Treatment categories**			
Farming alone (Fa_1_I_0_F_0_)	83.03	0	1
Farming and Informal Nonfarm (Fa_1_I_1_F_0_)	13.83	0	1
Farming and Formal Nonfarm (Fa_1_I_0_F_1_)	3.14	0	1
Total	100		
**Explanatory variables**			
Location (urban)	11.3	0	1
Gender (male)	79.3	0	1
Bank account ownership (%)	42.3	0	1
Credit access (%)	10.5	0	1
Remittances (%)	5.3	0	1
Polygamous family (%)	8.1	0	1

**Table 2b:** Descriptive statistics of explanatory variables – continuous variables.

Explanatory variables	Mean	SD	Min	Max
Age	48.426	14.607	17	97
Education	1.892	2.035	0	18
Education expenditure	1, 151.92	2,102.41	1	54, 019
Household size	5.313	3.289	1	28
**Farm characteristics**				
Farm size	6.773	6.568	0.5	50
Crop value	2,963.01	6285.611	0	168,000
Value of Agricultural Asset	333.8913	5618.978	0	25,500
Time spent on farming	32.965	16.488	1	98
Food consumption expenditure	1,571.123	1,513.424	10.160	19,048.01
Total observation	4120			

### 4.2 Determinant of participation in nonfarm activities

Given the multiple food security indicators (household food consumption expenditure, HDDS and FCS) used, six different results were obtained for the first stage of the METE; thus, factors affecting participation in nonfarm activities and the results are presented in Table 3. The base category was only farming household. The Wald chi^2^ value of 2440.28 and Prob > chi^2^ value of 0.0000 shows that at 1 % level of significance, all the coefficients of the covariates are significantly different from zero. According to the result, farming households within urban areas have higher likelihood to engage in informal nonfarm activities compared to rural households. The positive association between farmers’ geographical location and participation in nonfarm employment can be attributed to the wide range of nonfarm opportunities within urban areas for both skilled and unskilled farmers. Consistent with our findings, Diao et al. [82] reported that availability of ready market for nonfarm products or finished goods among urban areas may drive many urban households to engage in informal nonfarm activities. The insignificant effect of urban dwellers on formal nonfarm participation could be due to lack of the required skills among the farming households to engage in the sector. In addition, high population density and lack of space among many urban areas restricts many farming activities in these areas thereby causing residents to venture into nonfarm activities.

**Table 3:** Determinant of participation in nonfarm activities.

VARIABLES	HDDS		FCS		Consumption expenditure	
	Informal Nonfarm	Formal Nonfarm	Informal Nonfarm	Formal Nonfarm	Informal Nonfarm	Formal Nonfarm
Location (urban = 1)	**0.573**∗∗∗	0.386	**0.573**∗∗∗	0.386	**0.556**∗∗∗	0.381
	(0.156)	(0.282)	(0.156)	(0.282)	(0.157)	(0.282)
Gender (male = 1)	−1.564∗∗∗	0.444	**−1.563**∗∗∗	0.444	**−1.524**∗∗∗	0.443
	(0.151)	(0.316)	(0.151)	(0.316)	(0.151)	(0.316)
Age	**−0.032**∗∗∗	**−0.034**∗∗∗	**−0.032**∗∗∗	**−0.034**∗∗∗	**−0.030**∗∗∗	**−0.034**∗∗∗
	(0.004)	(0.007)	(0.004)	(0.007)	(0.004)	(0.007)
Marital Status	−0.040	−0.413	−0.040	−0.413	−0.026	−0.405
	(0.138)	(0.257)	(0.138)	(0.257)	(0.137)	(0.256)
Household size	0.025	0.051	0.025	0.051	0.017	0.050
	(0.021)	(0.037)	(0.021)	(0.037)	(0.023)	(0.037)
Education (Years)	**0.024**∗	0.034	**0.024**∗	0.034	**0.023**∗	0.033
	(0.014)	(0.022)	(0.014)	(0.022)	(0.014)	(0.022)
Farm Size (log)	−0.067	−0.122	−0.067	−0.122	−0.099	−0.125
	(0.068)	(0.112)	(0.068)	(0.112)	(0.069)	(0.112)
Credit Access (yes = 1)	0.056	**0.553**∗∗	0.057	**0.553∗∗**	0.048	**0.538∗**
	(0.171)	(0.277)	(0.171)	(0.277)	(0.169)	(0.278)
Remittances	**0.650**∗∗∗	**0.729**∗	**0.651**∗∗∗	0.729∗	**0.591**∗∗∗	**0.724**∗
	(0.218)	(0.399)	(0.218)	(0.399)	(0.219)	(0.400)
Total Crop Value (log)	−0.025	0.030	−0.025	0.030	−0.021	0.030
	(0.023)	(0.045)	(0.023)	(0.045)	(0.024)	(0.046)
Polygamous family	0.072	−0.223	0.071	−0.223	0.141	−0.218
	(0.246)	(0.451)	(0.246)	(0.451)	(0.237)	(0.445)
TLU	0.021	−0.090	0.021	−0.090	0.017	−0.089
	(0.016)	(0.060)	(0.015)	(0.060)	(0.015)	(0.061)
Forest Zone	**−0.896**∗∗∗	**−0.675**∗	**−0.896**∗∗∗	**−0.675**∗	**−0.873**∗∗∗	**−0.672**∗
	(0.191)	(0.348)	(0.191)	(0.348)	(0.188)	(0.348)
Savannah Zone	**−1.042**∗∗∗	**−0.622**∗	**−1.042**∗∗∗	**−0.622**∗	**−1.001**∗∗∗	**−0.612**∗
	(0.197)	(0.371)	(0.197)	(0.371)	(0.195)	(0.372)
Ownership of Bank Account	**0.739**∗∗∗	**0.527**∗∗	**0.741**∗∗∗	**0.527**∗∗	**0.770**∗∗∗	**0.547**∗∗
	(0.119)	(0.221)	(0.119)	(0.221)	(0.115)	(0.221)
Ownership of Phone	**0.755**∗∗∗	−0.000	**0.754**∗∗∗	−0.001	**0.813**∗∗∗	0.010
	(0.198)	(0.307)	(0.197)	(0.307)	(0.194)	(0.307)
Value Agricultural Asset	0.027	0.062	0.027	0.062	0.039	0.066
	(0.029)	(0.047)	(0.029)	(0.047)	(0.029)	(0.047)
Constant	0.189	−2.663∗∗∗	0.189	−2.662∗∗∗	0.039	−2.692∗∗∗
	(0.354)	(0.697)	(0.354)	(0.697)	(0.358)	(0.699)
					
Observations	4,120	4,120	4,120	4,120	4,120	4,120

Robust standard errors in parentheses.

∗∗∗p < 0.01, ∗∗p < 0.05, ∗p < 0.1.

Females have higher likelihood of engaging in informal nonfarm activities. Females are 1.57 times more likely to engage in informal nonfarm activities relative to male headed households. Previous studies mention that unavailability of productive assets or resource constraints such as smaller land size and unfavorable land tenure rights against females may compel women to engage in nonfarm work [83]. In a similar way, domestic roles often reduces the mobility of many females hence resort to nonfarm activities particularly household-based manufacturing and service activities (Haggblade et al. [84]. The insignificant effect of sex on households’ decision to participate in formal nonfarm activities may be due to the lack of time flexibility associated with the formal sector of which many farmers cannot be part due to their demanding roles.

The estimates indicate that age of household heads negatively affect farmers participation in formal and informal nonfarm economic activities. The negative coefficient of age implies older farmers are less likely to engage in both formal and informal nonfarm activities. Farming activities in many developing nations including Ghana are full of drudgery and combining it with other nonfarm activities put more labour burden on older farmers thereby reducing their likelihood of undertaking nonfarm activities. Younger farmers on the other hand are more energetic to combine farming with nonfarm activities. Sienso et al. [14] posited young farmers are more adventurous and inclined to change therefore can easily undertake nonfarm business.

There is positive effect of education on households’ decision to engage in informal nonfarm activities. This result was expected on the premise that education provides many people with the needed mental acumen required to manage their multiple investments especially nonfarm investments. Again, the higher income associated with higher education often creates a surplus funds for households to invest into nonfarm activities. Mathenge et al. [68] demonstrated that education of household head positive effect on salaried earnings and overall off farm earnings. However, education has no significant effect on formal nonfarm employment and it could be that although they are educated there is a mismatch between the knowledge acquired and the kind of formal employment farmers may want. This is in line with Readon et al. (2020) argument that the effect of education on nonfarm employment is sensitive to the schooling type and types of education.

Controlling for other factors, farmers who own bank account are 0.739 times more likely to participate in informal nonfarm activities and 0.527 more likely to undertake formal nonfarm activities. This means that farm households who own bank account can save with their banks to fund their business. In a similar way, ownership of bank account is a prerequisite factor for salary employment. In many Ghanaian formal sectors, payment through the banks is more common compared to face-to-face payment of employees. It is therefore not surprising that access to credit positively increases farmers’ chances of engaging in nonfarm employment.

Receipt of remittances increases the likelihood of engagement in informal nonfarm activities. Given the relatively high capital requirement to establish nonfarm businesses, many poor households may have to rely on external funding to establish theirs. Remittances from domestic and abroad relations provide support for resource constrained farmers to set up nonfarm business. For this reason, receipt of remittances is seen as an important factor that reduces the financial burden needed to undertake nonfarm activities. This is an indication that remittances received are channeled into nonfarm earning investment instead of consuming it (Dary and Ustarz 2020).

Farmers' agroecological location was found to have significant influence on farmers’ decision to participate in nonfarm activities. There is a negative association between households within the forest zone and participation in nonfarm activities. Thus, relative to coastal belt, farmers within the forest zone are less likely to engage in both formal and informal nonfarm activities. This can be attributed to the high value crops cultivated within the forest zones for which reasons farmers in the zone will have little desire to engage in nonfarm activities. In the Coastal zone where little cash crops are grown, Senadza [85] found that households earn more nonfarm income. Similarly, Savannah agroecological zone was statistically significant and negatively associated with participation in both formal and informal nonfarm activities. This implies that farming households within this zone tend to drift away from nonfarm activities consequently increasing their likelihood of undertaking farming activities more. This may be as result of the absence of vibrant nonfarm economic activities in the savannah areas of Ghana unlike the coastal belt. As a result, farming households within the Savanah belt will have little chance to engage in nonfarm employment. These findings confirm those of Canagarajah et al. [86].

ICT tools mainly phones are quick medium for accessing relevant information. Phones also offer farmers a medium for business ideas. The study finds a significant positive effect of ownership of phone on participation in nonfarm activities. From the study, we can deduce that mobile phone owners used their phones to maintain social networks and build business relations that positively impact their chances of undertaking informal nonfarm activities. The study did not observe any statistically significant effect of ownership of phones on participation in formal nonfarm activities. This could be that majority of those owning mobile phone do not have the skills that will give them the chance of securing a job in the formal sector.

### 4.3 Determinants of household food security

This section forms the second stage of the METE regression. The section analyzed the determinants of household food security using three (3) food security indicators (adult equivalent of per capita consumption expenditure, FCS and HDDS). The estimates reported in Table 4 reveal that households located in urban areas have higher food consumption expenditure, HDDS and FCS compared to rural households. This could be attributed to the high-income advantage urban households have over rural households. Urban households may have a higher income status as a result of more available economic activities that they undertake. This gives urban households a higher purchasing power to acquire more food. Tollens [87] explained that poverty is largely a rural phenomenon mostly in Sub-Sahara Africa which may account for the low food security among rural households. Furthermore, urban areas are often characterized by well-developed physical structures and transportation networks that support effective distribution of food to reach more consumers at lower cost which increases the food security status of urban households [88]. Additionally, urban centers provide larger markets for more diverse range of foods [89] to meet the varied tastes and nutritional needs of many urban consumers.

**Table 4:** Determinants of household food security.

Variables	Consumption Expenditure	FCS	HDDS
Location (urban = 1)	0.189∗∗∗	0.134∗∗∗	0.119∗∗∗
	(0.032)	(0.017)	(0.014)
Gender (Male = 1)	0.033	−0.102∗∗∗	−0.066∗∗∗
	(0.034)	(0.016)	(0.014)
Age	0.002∗∗∗	0.000	−0.001∗∗
	(0.001)	(0.000)	(0.000)
Marital status (1 = married)	−0.104∗∗∗	0.069∗∗∗	0.039∗∗∗
	(0.028)	(0.015)	(0.012)
Household size	−0.122∗∗∗	0.024∗∗∗	0.018∗∗∗
	(0.005)	(0.002)	(0.002)
Education (Years)	0.010∗∗∗	0.008∗∗∗	0.007∗∗∗
	(0.003)	(0.001)	(0.001)
Farm Size (log)	0.151∗∗∗	−0.019∗∗∗	−0.015∗∗∗
	(0.015)	(0.007)	(0.006)
Access to Credit (yes = 1)	0.137∗∗∗	0.131∗∗∗	0.085∗∗∗
	(0.039)	(0.017)	(0.013)
Remittance	−0.026	0.042∗	0.014
	(0.058)	(0.024)	(0.020)
Shock	−0.116	0.009	0.011
	(0.073)	(0.030)	(0.024)
Total Crop Value (log)	0.007	0.013∗∗∗	0.010∗∗∗
	(0.005)	(0.003)	(0.002)
Polygamous family	0.385∗∗∗	−0.019	−0.000
	(0.059)	(0.023)	(0.020)
Ownership of Fridge	0.242∗∗∗	0.138∗∗∗	0.112∗∗∗
	(0.038)	(0.019)	(0.014)
TLU	0.014∗∗∗	−0.000	0.001
	(0.004)	(0.002)	(0.001)
Forest Zone	0.142∗∗∗	−0.116∗∗∗	−0.150∗∗∗
	(0.041)	(0.020)	(0.015)
Savannah Zone	−0.331∗∗∗	−0.289∗∗∗	−0.212∗∗∗
	(0.043)	(0.021)	(0.016)
Constant	5.988∗∗∗	1.779∗∗∗	−0.510∗∗∗
	(0.123)	(0.047)	(0.039)
Lnα	1.341∗∗∗	1.922∗∗∗	2.284∗∗∗
	(0.081)	(0.035)	(0.030)
λ_1_ (Informal Nonfarm)	−0.475∗∗∗	−0.009∗∗∗	−0.006∗∗∗
	(0.035)	(0.002)	(0.002)
λ_2_ (Formal Nonfarm)	−0.133∗∗	−0.002∗∗	−0.001∗
	(0.063)	(0.001)	(0.001)

Robust standard errors in parentheses.

∗∗∗p < 0.01, ∗∗p < 0.05, ∗p < 0.1.

The result reveals a negative effect of sex on FCS and HDDS indicating that female-headed households are more food secured in terms of their FCS and HDDS. Females are more involved in food preparation activities, as such will put measures in place to ensure continuous food supply to their household members [90]. The findings concur with [91].

Age had varied effects on the various food security indicators. It positively influences food consumption expenditure but negatively associated with HDDS. The disparity may be s a result of the differences in the scale of measuring the food security indicators. The intuition behind these findings is that older people spend more of their income on food to gain some strength. However, the food they buy may not be able to meet their dietary needs. The insignificant effect of age on food consumption score implies that although households may spend on food items, the unit consumed may not be the desired level. The difference in the outcome brings to the fore the subjectivity associated with analyzing household food security. It is thus not surprising that although many empirical studies found age to negatively influence food security [[92], [93], [94]], other studies [57,62,63] have also found the opposite. Marital status (being married) is significant in explaining food security status of household members. Being married is negatively associated with food consumption expenditure but positively influence FCS and HDDS. The results suggest married household may spend what is enough to meet their food security needs and food requirements. The negative result found is consistent with the findings of [8]. The positive effect obtained between marital status and FCS and HDDS is consistent with the findings of Oyekale et al. [95].

Also, household size has a mixed effect on explaining the food security indicators. With regards to food consumption expenditure, household size is negatively related to food security. This implies that, at 1 % level of significance, an additional increase in household size by one person will decrease food consumption expenditure by Gh₵ 0.12 holding other factors constant. This corroborates the findings of [96]. However, it increases FCS and HDD. The mixed outcome can be as a result of the competing demand of meeting other basic needs of a large family for which reason a family may decide to buy food in bulk at a cheaper price. Hence, as household size increases they are still able to have a higher food consumption score and dietary diversity score.

An increase in years spent in formal education by one year will increase household food expenditure, FCS and HDDS by Gh₵ 0.01, 0.008 and 0.007 respectively. Generally, formal education improves people's nutritional knowledge and skills that results in increased food security at home. The result concurs with the recent study of [92]. The result indicates that at 1 % significance level, one percent increase in farm size will increase consumption expenditure by Gh₵ 0.151. This can be explained by highlighting the fact that when farm size increases households can obtain more output which underscores increased productivity. From the sales of output households get more income to spend on their food needs. Similar outcome has been reported by Adeniyi and Dinbabo [97] among smallholder irrigators in North West Nigeria. On the contrary, every percentage increase in farm size decreases the likelihood of a household being food secured by 0.019 point in terms of FCS and 0.015 points in terms of HDDS and vice versa. As unexpected as this may be a plausible explanation is that farmers who are constrained in terms of land tend to invest more in farm input and as such obtain a relatively higher mean yield compared to lager farm holdings (Headey et al. [98], this turns to improve household food security level.

Access to credit increases consumption expenditure by Gh₵ 0.137. In a similar way access to credit positively affect FCS and HDDS. This reflects the importance of credit mostly in a form of cash rather than farm inputs which gives households economic power to purchase more variety of food from local markets in order to meet their food needs. The results also buttress the point that households who have access to credit can smoothen their consumption across time and maintain a consumption level that is constant over a period. The studies of Zhao et al. [99] observed a positive effect of credit on HDDS and FCS. Again, households who receive remittances are 0.042 points more food secured in terms of FCS. Remittances are often seen as a supplementary income to increase households’ economic access to food thereby improving their food consumption [100].

A percentage increase in the total crop value leads to an increase in the FCS and HDDS by 0.013 and 0.010 scores respectively. Total crop value obtained is of great importance to household food security because higher crop value implies higher farm income. This high income enables farming households to obtain variety of food thereby reducing household vulnerability to food insecurity. Higher crop value in itself denotes higher farm output that will ensure food availability to households especially staples crops. Polygamous households spend Gh₵ 0.385 more on food expenditure compared to monogamous households. Polygamy among many Ghanaian societies is often characterized with high household size implying more mouths to be fed. However, more household members can imply more resources would be put together to acquire higher food consumption expenditure leading to improved household food security.

Many food preservation assets such as fridges extend the lifespan of food stuffs and also reduces food loses to ensure that food is made available to household for long period of time. According to the study, ownership of fridge positively and significantly affects household food consumption expenditure, FCS and HDDS. Households who own fridge have Gh₵ 0.242 more consumption expenditure and are more food secured in terms of FCS and HDDS by 0.138 and 0.112 points respectively. This is because by owing fridge households can buy in bulk for storage of variety of food stuffs to meet the food needs of the members. This is consistent with the findings of Zereyesus et al. [10]. At 1 % level of significance, increasing TLU by one unit will increase household consumption expenditure on food by Gh₵ 0.014. Previous studies explained that livestock are vital source of high valued protein such as meat, milk and eggs that plays an important role in ensuring food security [101]. The importance of livestock in improving food security can be seen through the sale of animals to increase household expenditure on foods.

Compared to costal ecological zones, households in forest zones have higher food consumption expenditure. This is in line with the GLSS7 report that indicated that expenditure on food among households within the forest zone is higher compared to coastal households. Conversely, the forest zone is negatively related to FCS and HDDS suggesting that household in the forest zones are less food secured in terms of FCS and HDDS. This suggests that farming households in the forest zone of Ghana seem to spend more on food but the quantity of food bought is unable to positively affect their FCS and HDDS. The savannah ecological zone is less food secured compared to coastal ecological households. The negative effect of savannah ecological zone on food security is similar across all the food security indicators and it can be attributed to the region being among the poorest region in the country and as such households may find it difficult to meet their food requirements. Consistent with the result of the GLSS7 report [102] poverty levels within the savannah areas are quite high and households spend relatively less on food as compared to households within the coastal zones. In addition, coastal zones exhibit bimodal rainfall pattern that enables households to farm twice in a year. This makes coastal households more advantageous in terms of crop production and farm income.

The study carefully examined the direction and the significance of the lambdas. All the factor loadings (k) reveals that there is evidence of negative selection bias signifying that unobserved factors increase the likelihood of participation in nonfarm activities are associated with lower levels of households' food security. The significant and negative value of the lambdas (λ) (informal nonfarm activities) suggests that at 5 % significant level, the unobservable characteristics of a farming household are associated with explaining farmers’ decision to participate in nonfarm activities. However, this effect has been controlled and corrected by the model which leads to unbiased estimates.

### 4.4 Impact of participation in nonfarm activities on food security

This section of the study presents the findings on the average treatment effect from the METE model. The findings are presented in Table 5. As expected, participation in nonfarm activities has a positive but different level of effect on all the food security indicators. Surprisingly, participation in informal nonfarm activities produces higher average effect compared to formal nonfarm activities. The average effect of participation in informal nonfarm activities increase per capital consumption expenditure by Gh₵ 0.504. In a similar way, participation in formal nonfarm activities also increases per capita consumption expenditure by Gh₵ 0.281. According to the study, participation in informal nonfarm activities have similar trend of higher average effect on household welfare compared to formal nonfarm activities with regards to FCS and HDDS. This then confirms the assertion made by Blekking et al. (2019) that within the African setting informal sector nonfarm participation can equally ensure food security to farmers.

**Table 5:** Average Treatment Effect of participation in Nonfarm Activities (METE).

Treatment Categories	Consumption Expenditure	HDDS	FCS
	ATE	ATE	ATE
Farming and informal nonfarm	0.504∗∗∗	0.080∗∗∗	0.086∗∗∗
	(0.043)	(0.013)	(0.016)
Farming and formal nonfarm	0.281∗∗∗	0.076∗∗∗	0.079∗∗∗
	(0.087)	(0.021)	(0.029)

Robust standard errors in parentheses∗∗∗p < 0.01, ∗∗p < 0.05, ∗p < 0.1.

To check for robustness, we estimated Propensity-score matching (PSM) in addition to the METE. Table 6 presents the results of the average treatment effect on the treated (ATT). The food security indicators were transformed (using natural log) in order to interpret the coefficients in terms of percentages. Four different matching algorithms or approaches (i.e Propensity-score matching (PSM), Inverse probability weighting (IPW), Inverse probability weighting with regression adjustment (IPWRA) and Nearest-neighbor matching (NNM)) were used in estimating the ATT in order to compare the robustness of the estimates. The results show that participation in informal nonfarm activities increases households’ per capita consumption expenditure on food by 9.7 %, HDDS by 4.6 % and FCS by 6 % while participation in formal nonfarm activities increases food security by 10 %, 8.2 % and 10.7 % for per capita consumption expenditure on food, HDDS and FCS respectively.

**Table 6:** Average Treatment Effect on the Treated of participation in Nonfarm Activities.

Estimation Strategy	Consumption Expenditure (log)	HDDS (log)	FCS (log)
	ATT	ATT	ATT
Informal Nonfarm Activities			
PSM	0.097∗	0.046	0.060
	(0.051)	0.020∗∗	0.026∗∗
IPW	0.067∗∗	0.056	0.045
	(0.033)	0.015∗∗∗	0.020∗∗
IPWRA	0.076∗∗	0.061	0.051
	(0.033)	0.015∗∗∗	0.020∗∗∗
NNM	0.70	0.059	0.049
	(0.044)	0.020∗∗∗	0.028∗
Formal Nonfarm Activities			
PSM	0.100	0.082∗	0.107
	(0.092)	(0.044)	(0.069)
IPW	0.142∗∗	0.077∗∗∗	0.069∗
	(0.061)	(0.025)	(0.037)
IPWRA	0.145∗∗	0.079∗∗∗	0.071∗∗
	(0.061)	(0.026)	(0.037)
NNM	0.084	0.087∗∗	0.092∗
	(0.084)	(0.040)	(0.051)

Robust standard errors in parentheses.

∗∗∗p < 0.01, ∗∗p < 0.05, ∗p < 0.1.

The estimation from the two methods conclusively shows that engaging in formal and informal nonfarm activities increases the food security of Ghanaian farmers. However, while the METE analysis shows greater effect comes from informal nonfarm activities, the ATT analysis shows the opposite results. It is an indication therefore that an analysis of this nature may be subjective to the analysis used. Researchers have demonstrated that nonfarm activities are important in reducing household vulnerability to food insecurity since they complement farm productivity through the investment of nonfarm income into farming businesses by purchasing farm inputs [[31], [32], [33]]. Similarly to our findings, Osarfo et al. [44] found that nonfarm activities increases household food security status Ghana.

## 5 Conclusion and recommendations

The study set out to investigate the impact of farmers’ engagement in nonfarm economic activities on the food security status of farming households. This was achieved using METE model. To this end, the study analyzed the determinants of formal and informal nonfarm activities and food security status of farming households and the contribution of formal and informal nonfarm activities on food security in Ghana. The results revealed that urban households, education and access to credit are some of the positive drivers of nonfarm activities while age and being a female reduce nonfarm activities. Food security is increased by income, remittance, owing a refrigerator and access to credit among other. However, leaving in the Savannah Zone of the country and a male reduces food security. It also found that by engaging in formal and informal nonfarm activities farm households make positive gains in per capita consumption, household dietary diversity and food consumption.

The implications from the findings include the need to pay attention to nonfarm economy as part of efforts to improve the welfare of farm households. Intervention in nonfarm economic activities should focus on programs that promote the development of agribusiness, small scale industry and agro based and non-agro based industries in order to facilitate the ease at which farming households can participate in nonfarm activities. With the coming on board of ACFTA and one district one factory (1D1F) provision of training to farmers may be beneficial to farmers who may want to engage in some activities after farming hours. Tailor made training for farmers business will also help them to manage their nonfarm business effectively and efficiently. The informal sector has the capability to promote food security of farm households. Policymakers need to therefore pay closer attention to this sector. As it is well known the sector is faced with a number of challenges. Addressing these challenges can therefore help in ensuring that households benefit much more.

## CRediT authorship contribution statement

**Eli Andrew Akosikumah:** Writing – original draft, Methodology, Investigation, Formal analysis, Data curation, Conceptualization. **Hamdiyah Alhassan:** Writing – review & editing, Validation, Supervision, Conceptualization. **Paul Adjei Kwakwa:** Writing – review & editing, Validation, Supervision.

## Data availability statement

Data will be made available on request. For requesting data, please write to the corresponding author.

## Declaration of competing interest

The authors declare that they have no known competing financial interests or personal relationships that could have appeared to influence the work reported in this paper.

## Figures and Tables

**Table:** 

HDDS	Farming and Informal	Farming and Formal
Joint significance of instruments	F(1,552) = 0.20	F(1, 111) = 4.35
**Consumption expenditure**		
Joint significance of instrument	F(1,552) = 0.19	F(1, 111) = 1.30
**FCS**		
Joint significance of instrument	F(1, 552) = 0.49	F(1, 111) = 1.43

## Appendix 1

Test of Validity of Instrument (Second Stage of METE) using Consumption Expenditure.

### Statements and Declarations

• Ethics approval and consent to participate: Not applicable
• Competing interests: The author declare that they have no competing interests
• Funding: Author did not receive any funding for this study
• Research involving Human Participants and/or Animals: Not applicable
• Informed consent: The study did not rely on data or participants that consent was needed.

## Footnotes

1 At the time of data collection, Ghana has 10 administrative regions but new regions have been created which brings the regions to 16.
